# Mitochondrial T16189C Polymorphism Is Associated with Metabolic Syndrome in the Mexican Population

**DOI:** 10.1155/2018/3981315

**Published:** 2018-03-25

**Authors:** Elsa Saldaña-Rivera, Marissa Jaqueline Careaga-Castilla, Giovani Daniel Olvera-Cárdenas, Elvia Pérez-Soto, Virginia Sánchez-Monroy

**Affiliations:** ^1^Laboratorio Multidisciplinario de Investigación, Escuela Militar de Graduados de Sanidad, Secretaría de la Defensa Nacional, CP 11200 Ciudad de México, Mexico; ^2^Facultad de Estudios Superiores Cuautitlán Izcalli, UNAM, CP 54740 Cuautitlán, Estado de México, Mexico; ^3^Universidad del Valle de México, Campus Lomas Verdes, CP 53220 Naucalpan de Juárez, Estado de México, Mexico; ^4^Secretaría de Investigación y Posgrado, Instituto Politécnico Nacional, CP 07738 Ciudad de México, Mexico; ^5^Laboratorio de Biomedicina Molecular I, Escuela Nacional de Medicina y Homeopatía, Instituto Politécnico Nacional, CP 07320 Ciudad de México, Mexico

## Abstract

Genetic factors, such as the mitochondrial DNA (mtDNA) T16189C polymorphism, have been associated with metabolic syndrome (MetS), but this association has not been studied in Mexico to date. The aim of the present study was to determine whether this polymorphism contributes to MetS in the Mexican population. We recruited 100 unrelated volunteer subjects who were divided into 2 groups: with MetS (MetS group) and without MetS (control group). All subjects were genotyped for the mtDNA T16189C polymorphism by polymerase chain reaction and sequencing. The mitochondrial T16189C polymorphism was detected in 24 (24%) of 100 subjects analyzed. The frequency of the mtDNA T16189C polymorphism was higher in the MetS group with 21 (32.3%) of 65 testing positive compared to 3 (8.5%) of 35 in the control group, indicating that this polymorphism is a probable risk factor for MetS in the Mexican population (odds ratio 5.0909, 95% CI 1.3977–18.5424, *P* = 0.0136). Our results may contribute to early diagnosis of MetS, which is essential for establishing changes in early stages of the disease to avoid further complications and pathologies, thereby preventing the development of type 2 diabetes and cardiovascular disease in Mexico.

## 1. Introduction

Metabolic syndrome (MetS) is a metabolic disorder that affects approximately 45% of the Mexican population according to the National Health and Nutrition Survey of 2012 [1]. The disorder involves a cluster of metabolic abnormalities, including centrally distributed obesity, decreased concentration of high-density lipoprotein cholesterol (HDL-C), elevated levels of triglycerides (TG), high blood pressure, and hyperglycemia [2]. These metabolic abnormalities directly increase the risk of developing cardiovascular disease and type 2 diabetes mellitus [3].

Genetic factors, including the mitochondrial DNA (mtDNA) T16189C polymorphism, have been associated with MetS and type 2 diabetes mellitus (DM2) [4–10].

T to C substitution at mtDNA position 16189, within the regulatory displacement loop (D-loop), produces an uninterrupted polycytosine stretch (poly-C) which interferes with mtDNA replication [10–12]. Because this variant maps precisely to the OriB origin of replication [13], Ye et al. renamed it the “OriB variant” [8]. In common with other homopolymeric C tracts in mtDNA [14], when the unbroken tract exceeds 11 bp, it generates heteroplasmic length variation. As well as this alteration, the mtDNA becomes less susceptible to DNase I, though this may be because of changes in DNA secondary structure or protein binding [10].

In Mexico, the association between MetS and the mtDNA T16189C polymorphism has not yet been studied. The aim of the present study was to determine whether this polymorphism contributes to MetS in the Mexican population.

## 2. Materials and Methods

### 2.1. Study Population

Our study was conducted in México City in the “Hospital Central Militar” of the National Defense Ministry of México. We recruited 100 unrelated volunteer subjects, who were divided into 2 groups: with MetS (MetS group) and without MetS (control group). The control group consisted of 35 healthy subjects, and the MetS group had 65 subjects diagnosed with MetS defined by the guidelines of the National Cholesterol Education Program Adult Treatment Panel III (NCEP/ATP III) most frequently used in the world and in medical practices [15]. Written informed consent was obtained from each participant. The Institutional Human Research Ethical Committee approved the protocol and informed consent forms.

### 2.2. Genotyping

Genomic DNA extracted with a DNeasy Blood and Tissue kit (Qiagen) from oral brushing was used as a template. Polymorphism detection was performed by polymerase chain reaction (PCR) with the specific primers 5′-CACCATTAGCACCCAAAGCT-3′ and 5′-GAGGATGGTGGTCAAGGGAC-3′. After PCR, products were purified using ExoSAP-IT (USB) and sequenced in an ABI PRISM 3130 automated DNA sequencer (Applied Biosystems) using an ABI PRISM BigDye Terminator v3.1 Cycle Sequencing Kit (Applied Biosystems).

### 2.3. Evaluation of Biochemical Parameters

Measurements of biochemical parameters were performed with an ADVIA 1800 Chemistry System (Siemens) from serum.

### 2.4. Statistical Analysis

All clinical data and biochemical parameters were expressed as the mean ± standard deviation (SD), and the comparison of variables between experimental groups was performed by Student's *t*-test. In addition, a one-way analysis of variance was conducted to determine the effect of the polymorphism on clinical variables. The chi-squared test was performed to categorical variables, and the odds ratio was determined with 95% confidence intervals as a measure of the association between polymorphism frequency and MetS. *P* value of <0.05 was considered to be statistically significant for all analysis

## 3. Results

Our study included 100 subjects, and the mean age of the participants was 58.35 ± 10.0; 78% of the study subjects were female, and only 22% were males. In this study, more than half of the study subjects (74%) are overweight (39%) and obese (35%). Overweight was present about 40% in both groups, and obesity was present in about 23% in the control group and 42% in the Mets group. The clinical characteristics of the study population are summarized in Table 1. According to the criteria by NCEP ATP III, abdominal obesity was present in 72% of the study subjects with about 51% in the control group and 83% in the Mets group; low HDL-C was present in 69% of the study subjects with about 31% in the control group and 83% in the Mets group; high TG was present in 36% of the study subjects with about 17% in the control group and 46% in the Mets group; blood pressure elevation was present in 29% of the study subjects with about 3% in the control group and 45% in the Mets group; and hyperglycemia was present in 40% of the study subjects with about 26% in the control group and 48% in the Mets group (Table 2).

**Figure 1:**
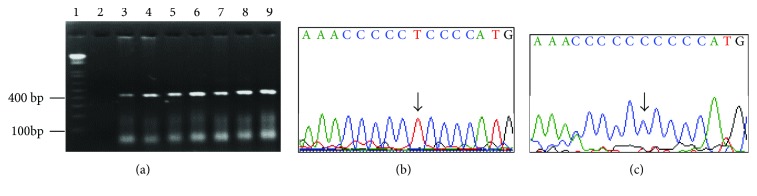
Detection of mitochondrial DNA (mtDNA) T16189 C polymorphism (a) 3% agarose gel (1) molecular marker, (2) no template PCR control, and (3–9) amplicons obtained from representative samples. (b, c) Wild-type sequence and mtDNA T16189C polymorphism, respectively. The arrows indicated the variants detected.

The mtDNA T16189C polymorphism was detected in 24 (24%) of 100 subjects analyzed. The frequency of the mitochondrial T16189C polymorphism was higher in the MetS group, with 21 (32.3%) of 65 testing positive compared to 3 (8.5%) of 35 in the control group, suggesting that the polymorphism is associated with MetS (odds ratio 5.0909, 95% CI 1.3977–18.5424, *P* = 0.0136).  Figure 1 shows representative sequences of the mitochondrial T16189C polymorphism detected.  Table 3 shows the clinical characteristics of subjects with or without the T16189C polymorphism, with observed differences in glycemia, triglycerides, and HDL-C and HBA1C values.

## 4. Discussion

In this work, we explored the mtDNA T16189C polymorphism in populations of subjects with or without MetS diagnosed based on NCEP/ATP III criteria. According to this definition, MetS is diagnosed if three of the five features are present; in our MetS group, 23% presented 4 o 5 features, which demonstrate an adequate grouping according to the NCEP/ATP III criteria. The study groups were homogeneous in terms of age and sex, which is very important because our population was of age > 40 years, and MetS has an increased risk in old age [16]. In addition, predominated by females, incidence of MetS differs between men and woman, which has been attributed to differences in risk factors and hormone production [17]. In this study, predominated overweight in the total population is in concordance with Mexican population data from the World Health Organization, which in 2016 reported that 57.6 to 70.7% of adults over 18 years were overweight [18]. With respect to NCEP/ATP III criteria, abdominal obesity (waist circumference) was the most frequent feature present in our MetS group and in total population, similar to a recent report, which indicated that abdominal obesity is a better indicator of metabolic risk in both genders in Mexico [19].

The mtDNA T16189C polymorphism explored here was detected in 25% of the study population, similar to the reported frequency in southern Brazil (21.15%) [20], less frequent than that in Asian populations (30%) [10], and more frequent than that in European populations (12.5%) [21]. The frequency similarity of our results with Brazil could be explained by similar phylogeny of the American population, as reported in findings described in meta-analysis from Europid populations, which suggest that the magnitude of association may vary by ethnic background [8].

We observed that the frequency of the mtDNA T16189C polymorphism was higher in the MetS group, with 21 (32.3%) of 65 testing positive compared to 3 (8.5%) of 35 in the control group, suggesting that this polymorphism is associated with MetS in the Mexican population as previously reported for other populations [5–7]. In addition, we detected changes in clinical parameters defined by MetS due to the presence of the mtDNA T16189C polymorphism, as evidenced by increased levels of glycemia, triglycerides, and HBA1C and decreased levels of HDL-C. Some authors have suggested that the loss of mitochondrial function, characterized by low expression of mtDNA and reduced levels of proteins involved in oxidative phosphorylation, might contribute to all components of metabolic syndrome [22, 23]. With respect to mtDNA T16189C, it has been reported that this polymorphism causes altered protein binding to the affected DNA region, subsequently leading to impaired mtDNA replication [10], while other reports suggest that the role of the mtDNA T16189C polymorphism probably involves the antioxidant defense system because of increased oxidative stress in diabetic patients [11]. In addition, it has been shown that subjects harboring the mtDNA T16189C polymorphism have altered the mtDNA copy number in blood cells [12]. However, more studies are required to confirm this polymorphism's biochemical effects on mitochondrial function.

Overweight and obesity are highly prevalent in the Mexican population. In addition, 25.2% of adults with Mets have a diagnosis of obesity, which is recognized as the main risk factor for MetS [1]. Furthermore, because MetS is associated with an increased risk of both type 2 diabetes and cardiovascular disease, two of the main causes of death in Mexico [24, 25], the results presented here may contribute to the early diagnosis of MetS. Early detection is essential for establishing changes in early stages of the disease to avoid further complications, thus preventing the development of diabetes and cardiovascular disease in Mexico.

In conclusion, our results indicate that the T16189C polymorphism is a possible risk factor for MetS in the Mexican population, but larger sample sizes are necessary to confirm the role of the susceptibility variant described in this report.

## Figures and Tables

**Table 1:** Characteristics of study population.

Characteristic	Control group	MetS group	*P* value
	Mean ± SD	Mean ± SD	
*n*	35	65	
Male/female	8/27	14/51	
Age (y)	58.40 ± 11.44	58.08 ± 9.24	0.879
Waist circumference (cm)	93.31 ± 9.22	99.78 ± 9.32	0.001
Body mass index (kg/m^2^)	26.41 ± 4.27	29.21 ± 5.07	0.006
Systolic blood pressure (mmHg)	107.83 ± 16.19	124.35 ± 18.37	<0.001
Diastolic blood pressure (mmHg)	70.20 ± 8.69	77.15 ± 8.29	<0.001
Glucose (mg/dl)	112.06 ± 63.53	135.29 ± 55.90	0.062
Triglycerides (mg/dl)	118.23 ± 43.56	168.37 ± 97.67	0.005
HDL-cholesterol (mg/dl)	51.12 ± 12.12	41.52 ± 8.72	<0.001
HBA1C (%)	6.55 ± 1.90	7.98 ± 2.40	0.003

**Table 2:** Presence of NCEP/ATPIII criteria in study population.

Criteria	Control group	MetS group	*P* value
	Mean ± SD	Mean ± SD	
Waist circumference			
>102 cm (males) >88 cm (females)	18/35 (51.43)	54/65 (83.08)	0.0004
HDL-C mg/dl			
<40 (males) <50 (females)	11/35 (31.43)	54/65 (83.07)	<0.0001
Triglycerides > 150 mg/dl	6/35 (17.14)	30/65 (46.15)	0.002
Blood pressure > 130/85 mmHg	1/35 (2.86)	29/65 (44.61)	<0.0001
Glucose > 110 mg/dl	9/35 (25.71)	31/65 (47.69)	<0.0152

**Table 3:** Characteristics of study population with and without mtDNA T16189C polymorphism.

Parameter	mtDNA T16189C polymorphism		
	T	C	*P*
*n*	76	24	
Male/female	15/61	7/17	
Age (y)	58.96 ± 10.69	55.75 ± 7.09	0.172
Waist circumference (cm)	97.38 ± 9.75	97.94 ± 9.90	0.809
BMI (kg/m^2^)	28.38 ± 5.01	27.77 ± 4.94	0.601
Systolic blood pressure (mmHg)	117.59 ± 17.92	121.67 ± 23.16	0.369
Diastolic blood pressure (mmHg)	74.50 ± 9.13	75.42 ± 8.84	0.667
Glycemia (mg/dl)	119.76 ± 54.37	150.58 ± 69.29	0.026
Triglycerides (mg/dl)	137.97 ± 62.17	191.50 ± 130.24	0.007
HDL-C (mg/dl)	46.88 ± 11.32	38.55 ± 6.86	<0.001
HBA1C (%)	7.15 ± 2.06	8.52 ± 2.84	0.012
